# Association between circadian variation of heart rate and mortality among critically ill patients: a retrospective cohort study

**DOI:** 10.1186/s12871-022-01586-9

**Published:** 2022-02-12

**Authors:** Jingjing Zhang, Linyun Du, Jiamei Li, Ruohan Li, Xuting Jin, Jiajia Ren, Ya Gao, Xiaochuang Wang

**Affiliations:** Department of Critical Care Medicine, the Second Affiliated Hospital of Xi’an Jiaotong University, Xi’an, China

**Keywords:** Circadian variation, Heart rate, Mortality, Intensive care unit

## Abstract

**Background:**

Heart rate (HR) related parameters, such as HR variability, HR turbulence, resting HR, and nighttime mean HR have been recognized as independent predictors of mortality. However, the influence of circadian changes in HR on mortality remains unclear in intensive care units (ICU). The study is designed to evaluate the relationship between the circadian variation in HR and mortality risk among critically ill patients.

**Methods:**

The present study included 4,760 patients extracted from the Multiparameter Intelligent Monitoring in Intensive Care II database. The nighttime mean HR/daytime mean HR ratio was adopted as the circadian variation in HR. According to the median value of the circadian variation in HR, participants were divided into two groups: group A (≤ 1) and group B (> 1). The outcomes included ICU, hospital, 30-day, and 1-year mortalities. The prognostic value of HR circadian variation was investigated by multivariable logistic regression models and Cox proportional hazards models.

**Results:**

Patients in group B (*n* = 2,471) had higher mortality than those in group A (*n* = 2,289). Multivariable models revealed that the higher circadian variation in HR was associated with ICU mortality (odds ratio [OR], 1.393; 95% confidence interval [CI], 1.112–1.745; *P* = 0.004), hospital mortality (OR, 1.393; 95% CI, 1.112–1.745; *P* = 0.004), 30-day mortality (hazard ratio, 1.260; 95% CI, 1.064–1.491; *P* = 0.007), and 1-year mortality (hazard ratio, 1.207; 95% CI, 1.057–1.378; *P* = 0.005), especially in patients with higher SOFA scores.

**Conclusions:**

The circadian variation in HR might aid in the early identification of critically ill patients at high risk of associated with ICU, hospital, 30-day, and 1-year mortalities.

**Supplementary Information:**

The online version contains supplementary material available at 10.1186/s12871-022-01586-9.

## Background

Heart rate (HR) is regulated by the autonomic nervous system, and therefore, the autonomic nervous system function could be reflected by HR variations, such as heart rate variability (HRV), heart rate turbulence (HRT), resting heart rate (RHR), and nighttime mean heart rate (NHR). Among them, HRV is measured by the variation in the beat-to-beat interval between consecutive heart beats to evaluate the autonomic nervous mediation of the cardiovascular system [1, 2]. Several studies have reported that the decrease in HRV was associated with impaired sympathetic modulation, which led to early in-hospital deterioration of sepsis and multiple organ dysfunction syndrome (MODS), and a higher mortality in the intensive care unit (ICU) [3–10]. However, Monfredi et al. have put forward a novel controversial discussion about the exponential decay relationship between HR and HRV, that is, HRV is primarily dependent on HR, with HRV increasing when HR slows, and vice versa [11]. Therefore, HRV cannot be used in any simple way to assess the autonomic nerve activity to the heart. HRT describes the physiological short-term oscillation of beat-to-beat intervals after spontaneous ventricular premature beats [12], which has been introduced as an autonomic predictor for cardiac mortality following myocardial infarction or heart failure [12, 13]. Besides, there have been previous reports in literature that increased resting HR can be associated with increased short-term and long-term mortality from critical illness [14–19]. Although the above parameters have been verified to have a higher predictive value for mortality, they either have their own limitations or ignore the circadian rhythm of the HR.

Most of physiological parameters, such as HR, blood pressure, spontaneous motor activity, cortisol, sleep cycles, the immune system, the autonomic nervous system, and body temperature follow 24-h stable circadian rhythms in healthy individuals [20]. However, these circadian rhythms may be interrupted or impaired by the loss of external zeitgebers when critically ill patients are often exposed to different degrees of artificial light, noise, continuous therapeutic intervention including ventilation, parenteral nutrition, and medications in the ICU [20–23]. Therefore, we took the circadian rhythms of HR into account to study the relationship between circadian variation of HR and mortality among critically ill patients admitted to the ICU in the Multiparameter Intelligent Monitoring in Intensive Care II (MIMIC-II) database.

## Methods

### Study design

This was an observational study conducted using individual medical information from the MIMIC-II database, which is maintained by the Laboratory for Computational Physiology at the Massachusetts Institute of Technology (MIT; Cambridge, MA) and contains de-identified data on patients hospitalized at an ICU at Beth Israel Deaconess Medical Center (Boston, MA) from 2001 to 2008 [24]. All procedures performed in studies involving human participants were in accordance with the ethical standards of the institutional research committee (Cardif Metropolitan University ethics committee—17/4/02R) and with the 1964 Helsinki Declaration and its later amendments or comparable ethical standards. Since the study did not impact clinical care and all protected health information was de-identified, the requirement for individual patient consent was waived by the Institutional Review Board of Beth Israel Deaconess Medical Center. All authors have attended the “protecting human subjects training” and obtained the certificate which permits access to the data.

### Date extraction

Patient data were exacted from MIMIC-II (version 2.6) using SAS version 9.4 (SAS Institute, Cary, NC). The extracted data, including patient identifiers, demographics, clinical diagnoses, medications effecting HR, scoring systems, length of stay in ICU, and complete HR records within the first 24 h after ICU admission, were acquired from 2001 to 2008 at Beth Israel Deaconess Medical Center. Age, sex, and ethnicity were included in the demographics and clinical diseases were diagnosed according to the International Classification of Diseases-9 diagnoses (ICD, 9th Editor), which includes respiratory failure, renal failure, liver cirrhosis, shock, diabetes uncomplicated, diabetes complicated, acquired immune deficiency syndrome (AIDS), lymphoma, metastatic cancer, coagulopathy, rheumatoid arthritis, infection, poisoning, hypoferric anemia, and a series of cardiovascular diseases. Severity-of-illness scores systems, including the Simplified Acute Physiology Score-I (SAPS-I) score and Sequential Organ Failure Assessment (SOFA) score, were calculated using physiological measurements and clinical information according to published recommendations and accepted formulae [25]. The medications included catecholamine, β-blockers, sedatives, and opioid analgesics.

### Patient selection

The data of a total of 4,760 adult patients were extracted from the MIMIC-II V2.6 database. These patients met the following characteristics: (1) single ICU admission; (2) the length of ICU stay ≥ 24 h; (3) no history of cardiovascular diseases, including cardiac arrhythmias, cardiac arrest, hypertension, heart failure, and coronary heart disease, or no use of cardiac pacemaker due to any cause; (4) complete HR and covariate records; (5) no outliers in HR records. Outliers were defined as values exceeding the mean ± 3 times the SD [26].

### Circadian rhythm of HR

HR was measured using a bedside monitor (Component Monitoring System IntelliVue MP-70; Philips Healthcare, Andover, MA) during the first 24 h in the ICU. In the present study, we focused on differences between the mean nighttime and daytime HR. Thus, the circadian rhythm of HR is calculated as the mean nighttime HR value divided by the mean daytime HR value. Patients were divided into two groups according to the median of the circadian rhythm of HR: the lower circadian rhythm of HR group (group A ≤ 1) and the higher circadian rhythm of HR group (group B > 1). The mean daytime HR value was computed as the average of all HR values from 7 am to 11 pm, and the mean nighttime HR was the average of all HR values from 11 pm to 7 am on the next day.

### Endpoints

The primary outcome measures in our study were ICU mortality and hospital mortality. The secondary outcome measures were 30-day mortality and 1-year mortality.

### Statistical analysis

Continuous variables are presented as the mean with SD or median with interquartile ranges. The one-way analysis of variance (ANOVA), or Kruskal–Wallis test was used as appropriate. Categorical variables are presented as frequency with percentage and compared using the Chi-Square test. To assess the association of circadian variation of HR with the study outcomes, we used univariate and multivariable logistic regression models for ICU and hospital mortality, cox proportional hazards model for the 30-day and 1-year mortality. All variables in the Cox proportional hazards model must meet the proportional hazard assumption. The multivariable models were adjusted for the following factors: (1) unadjusted in Model 1; (2) age, sex, and ethnicity in Model 2; (3) Model 2 plus clinical diagnoses (including respiratory failure, renal failure, liver cirrhosis, shock, diabetes uncomplicated, diabetes complicated, AIDS, lymphoma, metastatic cancer, coagulopathy, rheumatoid arthritis, infection, poisoning, and hypoferric anemia) in Model 3; (4) Model 3 plus medication taking on day 1 (including sedatives, catecholamines, β-blockers, and opioid analgesics) in Model 4; (5) Model 4 plus the first SOFA and SAPS-I score after ICU admission in Model 5; (6) Model 5 plus length of stay in ICU in Model 6; (7) Model 6 plus the first 24-h average HR in Model 7. Besides, based on the median of the first SOFA score, all patients were divided into the low SOFA score (0–5) group and the high SOFA score (6–24) group. Logistic regression and Cox regression were used to evaluate the relationship between the circadian rhythm of HR and outcomes in the two subgroups. *P* values of < 0.05 were considered to be statistically significant. All statistical analyses were performed with the SPSS software package version 22.0 (IBM Corp., Armonk, NY).

## Results

### Baseline characteristics

The records of 4760 patients were included in this study. Most of the study population came from surgical ICU (40.3%), followed by medical ICU (37.5%) (Supplementary Table 1). There were no significant differences in age, sex, ethnicity, and clinical diseases between the lower circadian rhythm of HR group (group A ≤ 1) and the higher circadian rhythm of HR group (group B > 1). However, patients in group B had higher SOFA scores [5.00 (2.00, 8.00) vs. 4.00 (2.00, 7.00), *P* = 0.001], SAPS-I scores [12.00 (9.00, 16.00) vs. 12.00 (8.00, 16.00), *P* = 0.006], and length of stay in ICU [2.52 (1.69, 4.96) vs. 2.31 (1.54, 4.26), *P* = 0.001) than those in group A. Moreover, patients in group B had a higher exposure rate for catecholamines (10.8% vs. 8.7%, *P* = 0.014) and opioid analgesics (2.5% vs. 1.3%, *P* = 0.002) than those in group A (Table 1). Besides, the univariate analysis of factors associated ICU, hospital, 30-day and 1-year mortality was presented in the Supplementary Table 2 and Supplementary Table 3. Furthermore, compared to ICU survival group, the dead patients during ICU stay were also more serious, with higher SOFA score [10.00 (7.00, 14.00) vs. 4.00 (2.00, 7.00), *P* < 0.001] and SAPS-I score [19.00 (15.00, 23.00) vs. 12.00 (8.00, 15.00), *P* < 0.001] (Table 2).

**Table 1:** Baseline Characteristics of the Study Subjects

Characteristics	Group A (≤ 1)	Group B (> 1)	*P* value
Subjects	2289	2471	
Age (≥ 65 yr), n (%)	594 (26.0)	662 (26.8)	0.511
Male, n (%)	1293 (56.6)	1363 (55.2)	0.346
Ethnicity, n (%)			0.295
White	1583 (69.2)	1664 (67.3)	
Asian	56 (2.4)	69 (2.8)	
Black	162 (7.1)	209 (8.5)	
Hispanic/Latino	100 (4.4)	96 (3.9)	
Unknown/Other	388 (17.0)	433 (17.5)	
Day 1 SOFA score	4.00 (2.00, 7.00)	5.00 (2.00, 8.00)	0.001
Day 1 SAPS-I score	12.00 (8.00, 16.00)	12.00 (9.00, 16.00)	0.006
Diagnosed by *ICD*, 9th Editor, n (%)			
Respiratory failure, n (%)	313 (13.7)	337 (13.6)	0.971
Renal failure, n (%)	364 (16.0)	486 (19.7)	0.001
Liver cirrhosis, n (%)	115 (5.0)	144 (5.8)	0.222
Shock, n (%)	142 (6.2)	152 (6.2)	0.940
Diabetes uncomplicated, n (%)	224 (9.8)	282 (11.4)	0.069
Diabetes complicated, n (%)	49 (2.1)	75 (3.0)	0.053
AIDS, n (%)	22 (1.0)	31 (1.3)	0.335
Lymphoma, n (%)	25 (1.1)	39 (1.6)	0.146
Metastatic cancer, n (%)	134 (5.9)	160 (6.5)	0.375
Coagulopathy, n (%)	129 (5.7)	167 (6.8)	0.109
Rheumatoid arthritis, n (%)	25 (1.1)	37 (1.5)	0.218
Infection, n (%)	235 (10.3)	282 (11.4)	0.204
Poisoning, n (%)	131 (5.7)	99 (4.0)	0.006
Hypoferric anemia, n (%)	231 (10.1)	271 (11.0)	0.327
Related medication taking in day 1, n (%)			
Sedatives, n (%)	42 (1.8)	67 (2.7)	0.043
Opioid analgesics, n (%)	29 (1.3)	61 (2.5)	0.002
Catecholamine, n (%)	199 (8.7)	267 (10.8)	0.014
β-blocking agent, n (%)	8 (0.3)	7 (0.3)	0.889
Length of stay in ICU, day	2.31 (1.54, 4.26)	2.52 (1.69, 4.96)	0.001
First 24-h mean HR	87.59 (77.11, 99.25)	88.78 (77.21, 100.14)	0.119
Mortality, n (%)			
ICU mortality, n (%)	151 (6.6)	238 (9.6)	< 0.001
Hospital mortality, n (%)	200 (8.7)	305 (12.3)	< 0.001
30-day mortality, n (%)	235 (10.3)	344 (13.9)	< 0.001
1-year mortality, n (%)	395 (17.3)	522 (21.1)	0.001
Follow time, day			
30-day mortality, day	27.97 ± 6.44	27.13 ± 7.60	< 0.001
1-year mortality, day	312.85 ± 119.66	299.84 ± 131.72	< 0.001

*SOFA* Sequential Organ Failure Assessment, *SAPS-I* Simplified Acute Physiology Score-I, *ICD* International Classification of Diseases, *AIDS* Acquired Immune Deficiency Syndrome

**Table 2:** Baseline Characteristics of the factors associated with ICU mortality and ICU survival

Characteristics	ICU mortality	ICU survival	*P* value
Subjects	389	4371	
Age (≥ 65 yr), n (%)	164 (42.2)	1092 (25.0)	< 0.001
Male, n (%)	214 (55.0)	2442 (55.9)	0.771
Ethnicity, n (%)			< 0.001
White	247 (63.5)	3000 (68.6)	
Asian	7 (1.8)	118 (2.7)	
Black	25 (6.4)	346 (7.9)	
Hispanic/Latino	5 (1.3)	191 (4.4)	
Unknown/Other	105 (27.0)	716 (16.4)	
Day 1 SOFA score	10.00 (7.00, 14.00)	4.00 (2.00, 7.00)	< 0.001
Day 1 SAPS-I score	19.00 (15.00, 23.00)	12.00 (8.00, 15.00)	< 0.001
Diagnosed by *ICD*, 9th Editor, n (%)			
Respiratory failure, n (%)	187 (48.1)	463 (10.6)	< 0.001
Renal failure, n (%)	172 (44.2)	678 (15.5)	< 0.001
Liver cirrhosis, n (%)	61 (15.7)	198 (4.5)	< 0.001
Shock, n (%)	86 (22.1)	208 (4.8)	< 0.001
Diabetes uncomplicated, n (%)	52 (11.3)	454 (10.4)	0.068
Diabetes complicated, n (%)	5 (1.3)	119 (2.7)	0.088
AIDS, n (%)	6 (2.8)	47 (1.2)	0.556
Lymphoma, n (%)	11 (12.6)	53 (5.6)	0.008
Metastatic cancer, n (%)	49 (5.9)	245 (6.5)	< 0.001
Coagulopathy, n (%)	48 (12.3)	248 (5.7)	< 0.001
Rheumatoid arthritis, n (%)	5 (1.3)	57 (1.3)	0.974
Infection, n (%)	43 (11.1)	474 (10.8)	0.899
Poisoning, n (%)	6 (1.5)	224 (5.1)	0.002
Hypoferric anemia, n (%)	35 (9.0)	467 (10.7)	0.298
Related medication taking in day 1, n (%)			
Sedatives, n (%)	21 (5.4)	88 (2.0)	< 0.001
Opioid analgesics, n (%)	20 (5.1)	70(1.6)	< 0.001
Catecholamine, n (%)	98 (25.2)	368 (8.4)	< 0.001
β-blocking agent, n (%)	1 (0.3)	12(0.3)	1.000
Length of stay in ICU, day	5.04 (2.31, 9.94)	2.30 (1.58,4.20)	< 0.001
Circadian variation of HR	1.00 (0.94, 1.06)	0.98 (0.93, 1.04)	< 0.001
First 24-h mean HR	94.45 (81.07, 107.39)	87.81 (76.94, 99.07)	< 0.001

*SOFA* Sequential Organ Failure Assessment, *SAPS-I* Simplified Acute Physiology Score-I, *ICD* International Classification of Diseases, *AIDS* Acquired Immune Deficiency Syndrome

### Association of the circadian variation of HR with mortality

**Fig. 1:**
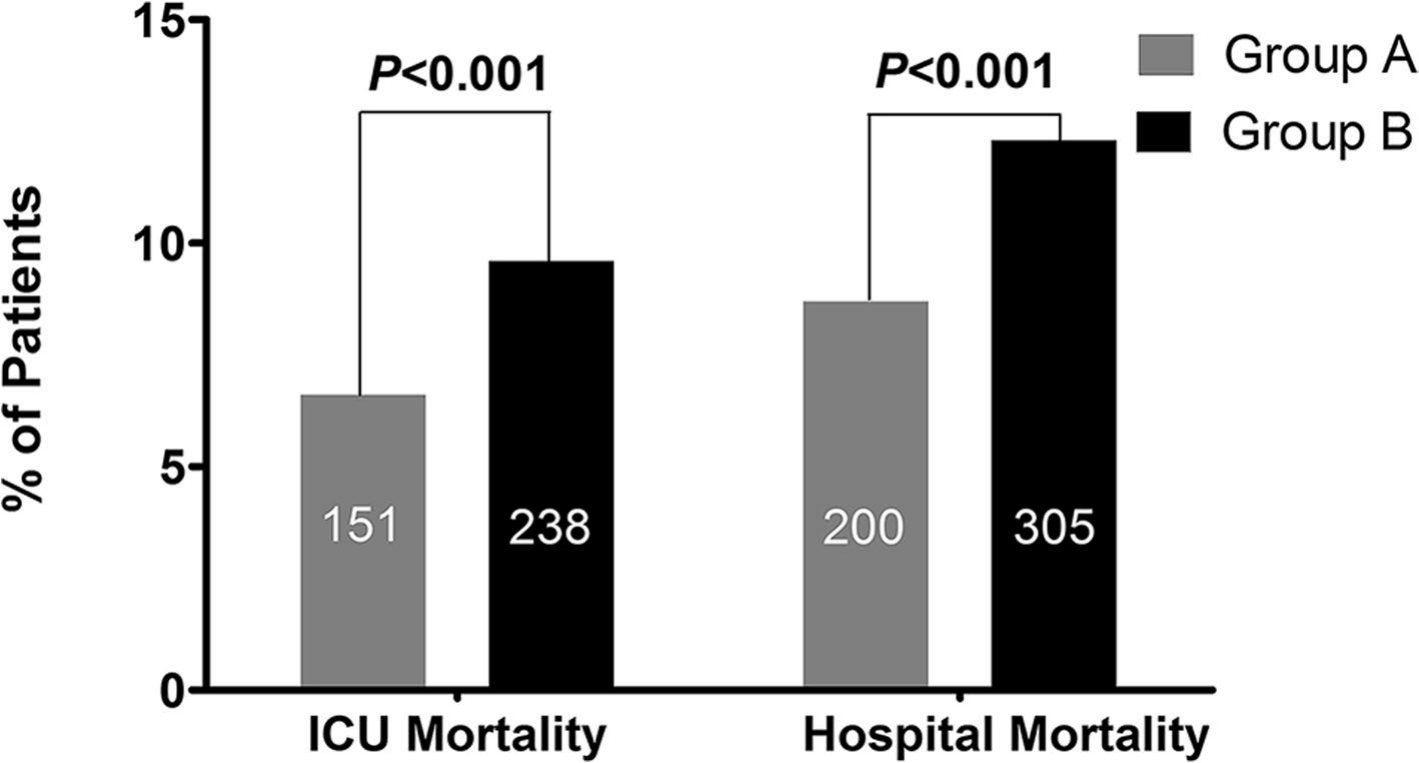
The difference in ICU and hospital mortality in patients with different circadian variation of HR. Patients in group B had higher ICU (*P* < *0.001*) and hospital mortality (*P* < *0.001*) than patients in group A

Patients in group B had higher ICU mortality (9.6% vs. 6.6%, *P* < 0.001), hospital mortality (12.3% vs. 8.7%; *P* < 0.001), 30-day mortality (13.9% vs. 10.3%; *P* < 0.001) and 1-year mortality (21.2% vs. 17.3%, *P* = 0.001) than that in group A patients (Table 1 and Fig. 1). These trends were also verified by the logistic regression analysis and the Cox regression model. Taking group A as the reference, after adjusting for all covariates, including age, sex, ethnicity, ICD-9 diagnoses, medication usage on day 1 of admission to the ICU, the first SOFA score, the first SAPS-I score, length of stay in the ICU, and the first 24-h average HR, the logistic regression results showed that group B, that is, the higher circadian variation of HR group was significantly associated with ICU mortality (odds ratio [OR], 1.393; 95% confidence interval [CI], 1.112–1.745; *P* = 0.004) and hospital mortality (OR, 1.393; 95% CI, 1.112–1.745; *P* = 0.004). Similarly, after adjusting for the same variables, the Cox proportional hazards model also showed that the higher circadian variation of HR group was significantly associated with 30-day mortality (hazard ratio, 1.260; 95% CI, 1.064–1.491; *P* = 0.007) and 1-year mortality (hazard ratio, 1.207; 95% CI, 1.057–1.378; *P* = 0.005) (Table 3).

**Table 3:** Association of circadian variation of heart rate with Mortality in Overall Patients

Logistic Regression	ICU mortality			Hospital mortality		
	OR	95% CI	*P*	OR	95% CI	*P*
Model 1	1.471	1.218–1.775	< 0.001	1.471	1.218–1.775	< 0.001
Model 2	1.463	1.210–1.768	< 0.001	1.463	1.210–1.768	< 0.001
Model 3	1.470	1.192–1.813	< 0.001	1.470	1.192–1.813	< 0.001
Model 4	1.417	1.146–1.752	0.001	1.417	1.146–1.752	0.001
Model 5	1.398	1.116–1.750	0.003	1.398	1.116–1.750	0.003
Model 6	1.397	1.115–1.748	0.004	1.397	1.115–1.748	0.004
Model 7	1.393	1.112–1.745	0.004	1.393	1.112–1.745	0.004
Cox Regression	30-day mortality			1-year mortality		
	HR	95% CI	*P*	HR	95% CI	*P*
Model 1	1.387	1.175–1.638	< 0.001	1.261	1.107–1.438	< 0.001
Model 2	1.376	1.165–1.624	< 0.001	1.254	1.100–1.429	0.001
Model 3	1.341	1.135–1.586	0.001	1.253	1.098–1.430	0.001
Model 4	1.307	1.105–1.546	0.002	1.233	1.081–1.408	0.002
Model 5	1.294	1.094–1.531	0.003	1.218	1.067–1.390	0.003
Model 6	1.277	1.079–1.510	0.004	1.212	1.062–1.383	0.004
Model 7	1.260	1.064–1.491	0.007	1.207	1.057–1.378	0.005

Model 1 unadjusted

Model 2 adjusted for age, gender, and ethnicity

Model 3 adjusted for Model 2 plus Respiratory failure, Renal failure, Liver cirrhosis, Shock, Diabetes uncomplicated, Diabetes complicated, AIDS, Lymphoma, Metastatic cancer, Coagulopathy, Rheumatoid arthritis, Infection, Poisoning, and Hypoferric anemia

Model 4 adjusted for Model 3 plus Medication taking in day 1 (including sedatives, catecholamine, β-blockers, Opioid analgesics)

Model 5 adjusted for Model 4 plus Sequential Organ Failure Assessment, and Simplified Acute Physiology Score-I

Model 6 adjusted for Model 5 plus length of stay in ICU

Model 7 adjusted for Model 6 plus average heart rate in the first 24 h

### Interaction analysis

Considering the SOFA score to be a strong predictor of mortality in ICU patients, the interaction effect between the circadian variation of HR and the SOFA score was also analyzed. The results revealed no interaction between them (*P* > 0.05), which verified the absence of synergistic or antagonistic effect between the two in the prediction of mortality and showed that they were independent of each other in predicting mortality.

### Sensitivity analysis

**Fig. 2:**
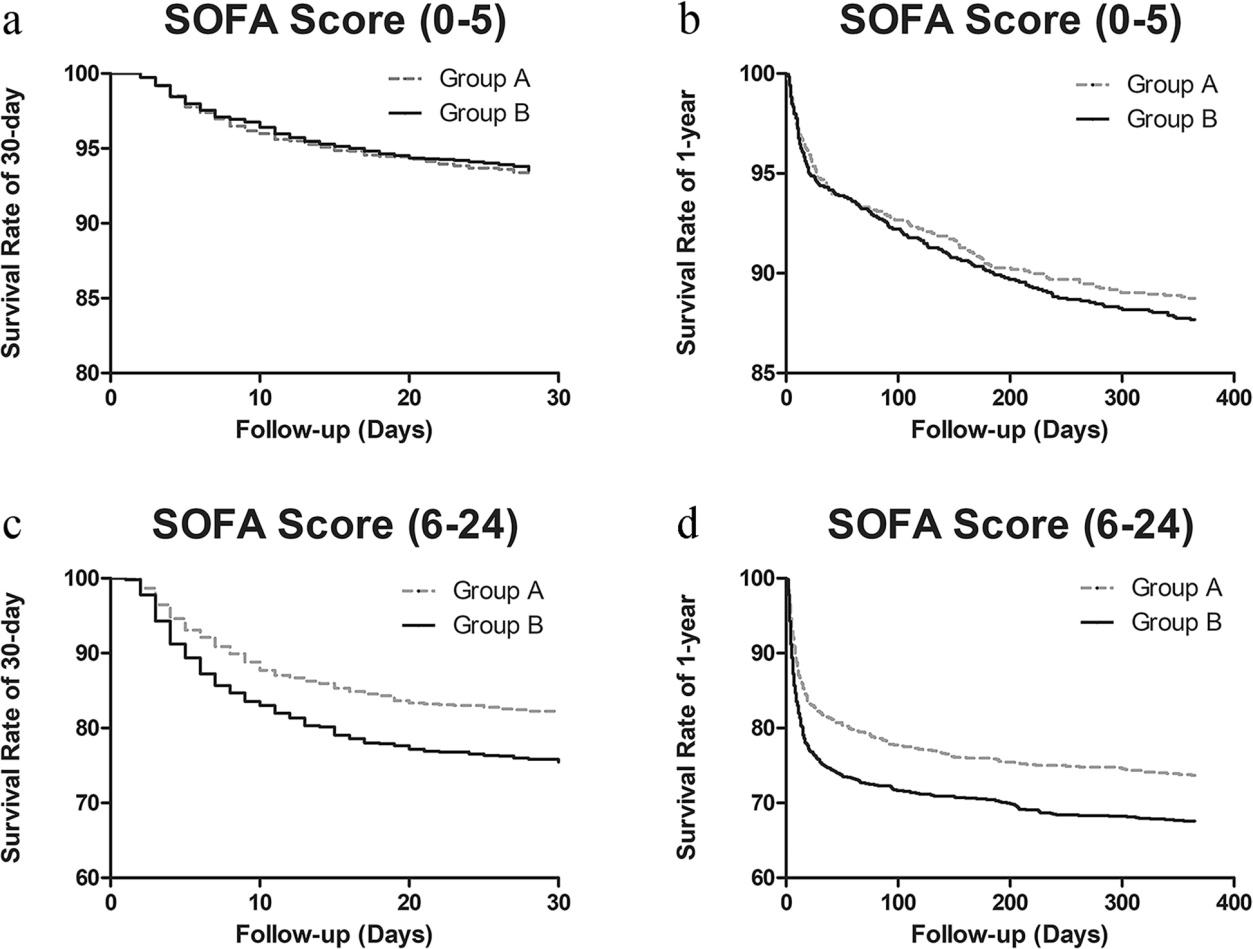
Kaplan–Meier survival analysis plot for mortality with different circadian variation of HR. Patients in group B had lower rates of 30-day **(c)** (*P* < *0.001*) and 1-year **(d)** (*P* < *0.001*) survival than patients in group A in the higher SOFA score subgroups. However, in the lower SOFA score subgroup, there was no statistical significance between the circadian variation of HR and 30-day (a) and 1-year (b) mortality

To diminish the influence of severity-of-illness on mortality from clinical practice, all individuals were also divided into two subgroups based on the median SOFA score of all the included patients: lower SOFA score group (0 ≤ SOFA score ≤ 5) and higher SOFA score group (6 ≤ SOFA score ≤ 24). Interestingly, in the higher SOFA score subgroup, taking group A as a reference, group B showed a remarkable association with short-term and long-term mortality in the multivariable regression model after adjusting a series of covariates. The specific statistical results were as follows: ICU mortality: OR, 1.444, 95% CI, 1.084–1.924, *P* = 0.012; hospital mortality: OR, 1.442, 95% CI, 1.110–1.873, *P* = 0.006; 30-day mortality: hazard ratio, 1.331, 95% CI, 1.091–1.624, *P* = 0.005; 1-year mortality: hazard ratio, 1.263, 95% CI, 1.069–1.493, *P* = 0.006. However, in the lower SOFA score subgroup, there was no statistical significance between the circadian variation of HR and mortality (Table 4). Kaplan–Meier survival curves also revealed that the short-term as well as long-term probability of survival was higher in group A than that in group B in the higher SOFA score subgroup (Fig. 2).

**Table 4:** Association of circadian variation of heart rate with Mortality in different SOFA score subgroups

	ICU mortality	Hospital mortality	30-day mortality	1-year mortality
SOFA score (0–5)	OR (95%CI)	OR (95%CI)	HR (95% CI)	HR (95% CI)
Model 1	1.180(0.703–1.980)	1.175(0.784–1.761)	1.078(0.782–1.484)	1.099(0.884–1.366)
Model 2	1.157(0.688–1.947)	1.155(0.769–1.735)	1.056(0.767–1.455)	1.086(0.874–1.350)
Model 3	1.385(0.794–2.416)	1.295(0.841–1.992)	1.113(0.806–1.538)	1.096(0.880–1.364)
Model 4	1.385(0.791–2.425)	1.280(0.830–1.975)	1.100(0.796–1.521)	1.086(0.872–1.353)
Model 5	1.482(0.813–2.642)	1.345(0.860–2.104)	1.108(0.799–1.535)	1.062(0.852–1.325)
Model 6	1.416(0.792–2.534)	1.304(0.833–2.043)	1.099(0.793–1.525)	1.049(0.840–1.308)
Model 7	1.534(0.848–2.777)	1.359(0.863–2.138)	1.162(0.836–1.617)	1.053(0.844–1.315)
SOFA score (6–24)	OR (95% CI)	OR (95% CI)	HR (95% CI)	HR (95% CI)
Model 1	1.493(1.171–1.903)	1.481(1.185–1.851)	1.436(1.181–1.745)	1.301(1.104–1.533)
Model 2	1.492(1.168–1.906)	1.483(1.184–1.857)	1.434(1.180–1.743)	1.306(1.108–1.539)
Model 3	1.480(1.134–1.932)	1.487(1.163–1.901)	1.414(1.160–1.722)	1.318(1.117–1.557)
Model 4	1.445(1.104–1.889)	1.453(1.135–1.861)	1.390(1.141–1.695)	1.296(1.097–1.531)
Model 5	1.453(1.091–1.935)	1.457(1.122–1.891)	1.380(1.132–1.682)	1.289(1.091–1.522)
Model 6	1.453(1.091–1.936)	1.457(1.122–1.891)	1.355(1.111–1.652)	1.274(1.078–1.504)
Model 7	1.444(1.084–1.924)	1.442(1.110–1.873)	1.331(1.091–1.624)	1.263(1.069–1.493)

Model 1 unadjusted

Model 2 adjusted for age, gender, and ethnicity

Model 3 adjusted for Model 2 plus Respiratory failure, Renal failure, Liver cirrhosis, Shock, Diabetes uncomplicated, Diabetes complicated, AIDS, Lymphoma, Metastatic cancer, Coagulopathy, Rheumatoid arthritis, Infection, Poisoning, and Hypoferric anemia

Model 4 adjusted for Model 3 plus Medication taking in day 1 (including sedatives, catecholamine, β-blockers, Opioid analgesics)

Model 5 adjusted for Model 4 plus Sequential Organ Failure Assessment, and Simplified Acute Physiology Score-I

Model 6 adjusted for Model 5 plus length of stay in ICU

Model 7 adjusted for Model 6 plus average heart rate in the first 24 h

## Discussion

In this study, we investigated the relationship between circadian variation of HR and mortality in ICU patients. Our results show that patients with a higher circadian variation of HR had higher ICU mortality, hospital mortality, 30-day mortality and 1-year mortality than that in patients with a lower circadian variation of HR after adjusting for demographic parameters, clinical comorbidities, medications affecting heart rate, severity scoring systems including SAPS-I and SOFA score, length of stay in the ICU, and the first 24-h average HR.

A circadian pattern was observed in the variation of many biological and psychological processes, most of which comply a rhythmically change with a period length of approximately 24 h. Previous studies have reported the existence of circadian variations in cognitive functions [27, 28] as well as the underlying brain processes [29] in healthy individuals. Recently, there’s growing interest in the research of disturbed circadian rhythms in patients situated in special environments settings without clearly delineated day or night, such as ICUs or long-term care homes [23, 30, 31]. Patients from ICUs commonly suffer from impaired sleep quality, which may result from the unnatural light rhythm and light intensity, further leading to impaired immune responses and ability of recovery from illness [23, 32], ultimately causing different prognosis.

Paul and Lemmer showed that the 24-h profile parameters, including cortisol, blood pressure, HR, body temperature, and spontaneous motor activity, were all disturbed in 24 critically ill sedated patients compared with the normal rhythmic 24-h patterns in healthy controls [21]. However, clinical complications that occur when circadian rhythms are disturbed have been generally unappreciated, especially in ICU patients. Therefore, our team has been working on the study of the influence of circadian rhythms of physiological signals on the prognosis of ICU patients. Previous research has found that reduced circadian fluctuations of mean arterial pressure were associated with hospital mortality and ICU mortality among critically ill patients [33]. Besides, we also found that rise in the nocturnal mean arterial pressure within the first 24 h of admission might serve as an independent risk factor for short-term and long-term mortality; this may help in early risk stratification and personalized mean arterial pressure management in the ICU [34].

Previous studies have reported multiple HR variations. Among them, HRV, a temporal beat-to-beat variation in successive RR intervals on an electrocardiographic (ECG) recording, was considered as a reflection of the regulation of HR by the autonomic nervous system [35]. In recent years, the prognostic value of HRV was evaluated outside a specific cardiological context. The reduction in HRV was associated with a higher mortality in critically ill patients as reported in several studies [36]. More specifically, descending HRV was found as a common feature in septic patients. Especially, impaired sympathetic modulation, represented by low frequency power in spectrum analysis of HRV, could be the early marker of MODS for ICU patients with sepsis [8]. In addition, the low-/high-frequency power ratio in spectrum analysis of HRV, considered as a representation of sympathovagal imbalance, may predict early clinical deterioration [9], providing an early indication for the identification of impending septic shock among emergency department patients [10]. In the meantime, upon ICU admission, a short-term reduction of HRV was also reported as a predictive factor for the mortality in septic patients [37].

HRT is another parameter that describes the physiological short-term oscillation of beat-to-beat intervals after spontaneous ventricular premature beats [38]. HRT also could independently predict cardiac mortality in patients with diabetes mellitus or simple ischemic heart disease [12, 39]. Notably, a meta-analysis reveals that elevated resting HR, an indicator of myocardial oxygen consumption, may increase the mortality of acute coronary syndrome patients in the coronary intervention era [40]. Besides, a cohort study on the Japanese general population reports that nighttime mean HR is associated with increased risk of all-cause mortality in epidemiological settings [18]. Furthermore, by using pervasive sensing technology and artificial intelligence in ICU, the autonomous and granular monitoring system presented that the delirious patients in ICU had higher average heart rate [41]. These indices can be easily calculated from a 24-h Holter ECG. An increasing amount of evidence also supports the association of these parameters with adverse outcomes [42].

In this study, the circadian variation in HR was proposed as a powerful parameter for assessing HR change and emphasizes the circadian rhythm, which should be able to better predict the occurrence of adverse events combined with previous parameters in critically ill patients. In normal conditions, the circadian decline rate of HR is within 10%-20%, called the nocturnal dipping of HR, and is mostly determined by the endogenous neuroendocrine rhythm [18, 43]. Critically ill patients experience circumstance alterations in light–dark cycles, auditory disruption, iatrogenic treatment, and psychological reactions when they are admitted to the ICU. These external changes disturb the endogenous neuroendocrine rhythm and sleep–wake cycle, which may contribute to the nocturnal non-dipping or anti-dipping of HR. The nighttime higher HR or reduced dipping of sleep HR can represent the status of sympathetic overdrive. On the one hand, higher nighttime HR caused by sympathetic overdrive could be associated with insulin resistance or direct atherosclerotic lesions via hemodynamic disturbances [44, 45]. On the other hand, sleep HR was a better marker of mortality than awake or clinical HR [46]. Therefore, we proposed that circadian variations of HR (calculated as the ratio of NHR to daytime mean HR) reflected impaired autonomic nerve regulation. Abnormal circadian variation of HR might be associated with adverse prognosis.

The SOFA score is an acknowledged parameter that is robustly correlated with patient outcomes in the ICU setting [25]. Therefore, we also conducted a sensitivity analysis in which the patients were divided into two subgroups according to the median of SOFA scores. Surprisingly, the association between circadian variation of HR and mortality persisted in the higher SOFA score subgroup but not in the lower after adjusting for a series of variables. The regression analysis verified the association of circadian variation of HR with mortality again. Therefore, a higher circadian fluctuation of HR might serve as an independent risk factor for both short-term mortality and long-term mortality in critically ill patients, especially in the higher SOFA score individuals.

The main strength of our study is the high number of general ICU patients included. To our knowledge, this is the first report that circadian variation of HR is associated with short-term mortality and long-term mortality in ICU patients. The results reveal the importance of circadian rhythm in ICU patient management, which reminds the clinicians to attach great importance to the changes of the circadian rhythm parameters in ICU patients and reduce the external disturbance at nighttime; besides, considering that cardiovascular disease could have a significant effect on HR or HR variations, and the mechanism between them has been not well understood, we excluded these patients to reduce interference of confounding factors. It is important to carry out risk stratification in the early stage to reduce the mortality rate and improve the prognosis of ICU patients.

Several limitations of this study warrant discussion. Firstly, limited by the observational design, causal conclusions cannot be reached. However, a wide range of clinically important confounders were included to reach valid results. Secondly, based on data from a single academic medical center, the applicability of our findings was limited in other sites. Thirdly, confined by the availability of data, SAPS I score, instead of SAPS II, was adopted in the present study in assessing the disease severity along with SOFA score. Fourthly, the MIMIC II database is relatively old. We will continue to mine the updated MIMIC database in the subsequent study to further verify our research results. Finally, as limitations of a retrospective study, undetected potential confounders may also exist. Although multivariable models were adjusted for as many confounding factors as possible to diminish the possible influences, residual confounding factors may still exist and need to be investigated in the future.

## Conclusions

The present study revealed the short-term and long-term prognostic value of the higher circadian variation of HR among critically ill patients, especially in individuals with higher SOFA scores. Therefore, the circadian variation of HR, as a cost-effective and readily available parameter, may serve as a potentially indicator of prognosis in the management of ICU patients. For the clinicians, further management, such as reducing the number of operations, reducing ward noise, or lowering light intensity level at night, may be considered to improve the circadian HR rhythm of ICU patients to improve their prognosis. Certainly, our findings are also needed to be confirmed by future studies, especially large prospective studies with a longer follow-up.

## Supplementary Information

**Additional file 1: Table 1.** The ICU admission types of the present study subjects.

**Additional file 2: Table 2.** The univariate analysis of factors associate ICU, hospital mortality in the wholepopulations.

**Additional file 3: Table 3.** The univariate analysis of factors associate 30-day and1-year in the whole populations.

## Data Availability

The datasets used and/or analyzed during the current study are available from the corresponding author on reasonable request.

## Abbreviations

AIDS: Acquired immune deficiency syndrome
CI: Confidence interval
ECG: Electrocardiographic
HR: Heart rate
HRT: Heart rate turbulence
HRV: Heart rate variability
ICD: International Classification of Diseases-9 diagnoses
ICU: Intensive care units
MIMIC-II: Multiparameter Intelligent Monitoring in Intensive Care II
MIT: Massachusetts Institute of Technology
MODS: Multiple organ dysfunction syndrome
NHR: Nighttime mean heart rate
OR: Odds ratio
RHR: Resting heart rate
SAPS-I: Simplified Acute Physiology Score-I
SD: Standard deviation
SOFA: Sequential Organ Failure Assessment
