# Supplementary material for: Association between circadian variation of heart rate and mortality among critically ill patients: a retrospective cohort study
Source: BMC Anesthesiol. 2022 Feb 12;22:45. doi: 10.1186/s12871-022-01586-9 (PMC8840314; doi:10.1186/s12871-022-01586-9)
Supplement: Supplementary file 1 — Additional file 1: Table 1. The ICU admission types of the present study subjects. [file 12871_2022_1586_MOESM1_ESM.docx]

Supplementary Table 1 The ICU admission types of the present study subjects

|  | MICU | SICU | CCU | CSRU | Others |
| --- | --- | --- | --- | --- | --- |
| Group A | 867(37.9%) | 929(40.6%) | 156(6.8%) | 337(11.1%) | 83(3.6%) |
| Group B | 919(37.2%) | 987(39.9%) | 171(6.9%) | 309(12.5%) | 85(3.4%) |
| Total | 1786(37.5%) | 1916(40.3%) | 327(6.9%) | 563(11.8%) | 168(3.5%) |

medical ICU (MICU), surgical ICU (SICU), cardiac (CCU), cardiac surgery recovery (CSRU)
