# Supplementary material for: Association between circadian variation of heart rate and mortality among critically ill patients: a retrospective cohort study
Source: BMC Anesthesiol. 2022 Feb 12;22:45. doi: 10.1186/s12871-022-01586-9 (PMC8840314; doi:10.1186/s12871-022-01586-9)
Supplement: Supplementary file 2 — Additional file 2: Table 2. The univariate analysis of factors associate ICU, hospital mortality in the wholepopulations. [file 12871_2022_1586_MOESM2_ESM.docx]

Supplementary Table 2 The univariate analysis of factors associate ICU, hospital mortality in the whole populations

|  | ICU mortality | | Hospital mortality | |
| --- | --- | --- | --- | --- |
|  | OR (95%CI) | *P* Value | OR (95%CI) | *P* Value |
| HRV group | 1.509(1.220-1.866) | <0.001 | 1.471(1.218-1.775) | <0.001 |
| age | 2.189(1.770-2.707) | <0.001 | 2.163(1.788-2.617) | <0.001 |
| gender | 1.031(0.837-1.271) | 0.771 | 1.005(0.834-1.210) | 0.961 |
| ethnicity | 1.116(1.050-1.187) | <0.001 | 1.090(1.031-1.153) | 0.002 |
| Respiratory failure | 7.814(6.264-9.746) | <0.001 | 6.305(5.150-7.721) | <0.001 |
| Renal failure | 4.323(3.482-5.368) | <0.001 | 4.175(3.431-5.079) | <0.001 |
| Liver cirrhosis | 3.920(2.880-5.335) | <0.001 | 4.026(3.029-5.351) | <0.001 |
| Shock | 5.681(4.306-7.494) | <0.001 | 5.356(4.125-6.954) | <0.001 |
| Diabetes uncomplicated | 1.331(0.978-1.811) | 0.069 | 1.206(0.908-1.601) | 0.195 |
| Diabetes complicated | 0.465(0.189-1.145) | 0.096 | 0.349(0.142-0.857) | 0.022 |
| AIDS | 1.441(0.612-3.391) | 0.403 | 1.080(0.460-2.539) | 0.860 |
| Lymphoma | 2.370(1.228-4.576) | 0.010 | 2.186(1.180-4.047) | 0.013 |
| Metastatic cancer | 2.427(1.751-3.362) | <0.001 | 2.493(1.855-3.350) | <0.001 |
| Coagulopathy | 2.340(1.685-3.249) | <0.001 | 2.305(1.708-3.112) | <0.001 |
| Rheumatoid arthritis | 0.985(0.393-2.472) | 0.974 | 0.905(0.388-2.110) | 0.817 |
| Infection | 1.022(0.734-1.423) | 0.899 | 0.981(0.728-1.321) | 0.898 |
| Poisoning | 0.290(0.128-0.657) | 0.003 | 0.292(0.144-0.596) | 0.001 |
| Hypoferric anemia | 0.826(0.576-1.185) | 0.299 | 0.883(0.645-1.207) | 0.434 |
| sedatives | 2.777(1.705-4.523) | <0.001 | 2.586(1.638-4.082) | <0.001 |
| catecholamine | 3.663(2.846-4.716) | <0.001 | 3.280(2.593-4.148) | <0.001 |
| β-blockers | 0.936(0.121-7.219) | 0.950 | 0.702(0.091-5.407) | 0.734 |
| Opioid analgesics | 3.330(2.003-5.536) | <0.001 | 3.357(2.097-5.376) | <0.001 |
| SOFA group | 9.090(6.845-12.072) | <0.001 | 6.975(5.547-8.771) | <0.001 |
| SOFA score | 1.368(1.330-1.407) | <0.001 | 1.334(1.301-1.368) | <0.001 |
| SAPS-I scores | 1.275(1.247-1.305) | <0.001 | 1.254(1.229-1.279) | <0.001 |
| Length of stay in ICU | 1.052(1.040-1.064) | <0.001 | 1.050(1.039-1.061) | <0.001 |
| First 24-hour average HR | 1.025(1.018-1.031) | <0.001 | 1.024(1.018-1.030) | <0.001 |
