# Supplementary material for: Association between circadian variation of heart rate and mortality among critically ill patients: a retrospective cohort study
Source: BMC Anesthesiol. 2022 Feb 12;22:45. doi: 10.1186/s12871-022-01586-9 (PMC8840314; doi:10.1186/s12871-022-01586-9)
Supplement: Supplementary file 3 — Additional file 3: Table 3. The univariate analysis of factors associate 30-day and1-year in the whole populations. [file 12871_2022_1586_MOESM3_ESM.docx]

Supplementary Table 3 The univariate analysis of factors associate 30-day and 1-year in the whole populations

|  | 30-day mortality | | 1-year mortality | |
| --- | --- | --- | --- | --- |
|  | HR (95%CI) | *P* Value | HR (95%CI) | *P* Value |
| HRV group | 1.387(1.175-1.638) | <0.001 | 1.261(1.107-1.438) | <0.001 |
| age | 2.245(1.905-2.646) | <0.001 | 2.689(2.362-3.062) | <0.001 |
| gender | 1.009(0.856-1.189) | 0.913 | 0.996(0.874-1.135) | 0.953 |
| ethnicity | 1.078(1.027-1.133) | 0.003 | 1.056(1.015-1.099) | 0.007 |
| Respiratory failure | 4.582(3.875-5.419) | <0.001 | 3.482(3.025-4.009) | <0.001 |
| Renal failure | 3.633(3.078-4.288) | <0.001 | 3.071(2.683-3.516) | <0.001 |
| Liver cirrhosis | 3.325(2.641-4.186) | <0.001 | 3.038(2.500-3.692) | <0.001 |
| Shock | 3.893(3.150-4.810) | <0.001 | 3.020(2.502-3.645) | <0.001 |
| Diabetes uncomplicated | 1.285(1.009-1.635) | 0.042 | 1.363(1.128-1.646) | 0.001 |
| Diabetes complicated | 0.377(0.169-0.842) | 0.017 | 0.753(0.478-1.186) | 0.221 |
| AIDS | 0.944(0.422-2.109) | 0.887 | 1.180(0.667-2.085) | 0.570 |
| Lymphoma | 2.079(1.245-3.472) | 0.005 | 2.216(1.477-3.323) | <0.001 |
| Metastatic cancer | 3.073(2.454-3.849) | <0.001 | 4.316(3.653-5.099) | <0.001 |
| Coagulopathy | 1.839(1.410-2.398) | <0.001 | 1.937(1.569-2.391) | <0.001 |
| Rheumatoid arthritis | 0.932(0.442-1.963) | 0.853 | 0.828(0.444-1.544) | 0.552 |
| Infection | 0.655(0.482-0.891) | 0.007 | 0.947(0.767-1.168) | 0.610 |
| Poisoning | 0.298(0.154-0.575) | <0.001 | 0.346(0.214-0.558) | <0.001 |
| Hypoferric anemia | 0.956(0.730-1.251) | 0.741 | 1.035(0.841-1.274) | 0.744 |
| sedatives | 2.563(1.775-3.702) | <0.001 | 2.241(1.632-3.077) | <0.001 |
| catecholamine | 2.603(2.126-3.186) | <0.001 | 2.538(2.153-2.992) | <0.001 |
| β-blockers | 1.252(0.312-5.016) | 0.751 | 2.046(0.850-4.928) | 0.110 |
| Opioid analgesics | 3.077(2.105-4.498) | <0.001 | 2.655(1.911-3.689) | <0.001 |
| SOFA group | 4.367(3.626-5.260) | <0.001 | 2.889(2.524-3.308) | <0.001 |
| SOFA score | 1.233(1.213-1.253) | <0.001 | 1.188(1.171-1.205) | <0.001 |
| SAPS-I scores | 1.193(1.176-1.209) | <0.001 | 1.152(1.139-1.165) | <0.001 |
| Length of stay in ICU | 1.018(1.009-1.028) | <0.001 | 1.025(1.019-1.032) | <0.001 |
| First 24-hour average HR | 1.021(1.016-1.026) | <0.001 | 1.011(1.007-1.015) | <0.001 |
